# Analysis of Immunohistochemical Expression of Bcl-2 in Cases of Psoriasis and Psoriasiform Dermatitis

**DOI:** 10.7759/cureus.71702

**Published:** 2024-10-17

**Authors:** Yazhini Chandrasekaran, Balaji Radhakrishnan, Shivasekar Ganapathy

**Affiliations:** 1 Department of Pathology, Sri Ramaswamy Memorial (SRM) Medical College Hospital and Research Centre, Sri Ramaswamy Memorial Institute of Science and Technology (SRMIST), Chengalpattu, IND

**Keywords:** apoptosis in psoriasis, bcl-2 in psoriasis and psoriasiform dermatitis, psoriasiform dermatitis, psoriasiform dermatoses, psoriasis

## Abstract

Introduction

Psoriasis involves rapid cell growth and abnormal differentiation of skin cells. Dysregulation of apoptosis has been proposed in the pathogenesis of psoriasis. The role of Bcl-2 as an anti-apoptotic protein in pathogenesis has not been studied in detail in these cases. Our study aims to evaluate the expression of Bcl-2 in different skin compartments in psoriasis and psoriasiform dermatitis cases.

Materials and methodology

An analytical cross-sectional study was performed using paraffin blocks of psoriasis and psoriasiform dermatitis patients between 2022 and 2023. For light microscopy screening, sections were initially stained with hematoxylin and eosin stains. In further sections, a primary antibody against Bcl-2 was applied. The level of Bcl-2 expression in keratinocytes of supra-basal and basal epidermal layers and dermal lymphocytes was evaluated using a scoring system.

Results

Sixty patients, 30 with psoriasis and 30 with psoriasiform dermatitis were included. Among the histopathological features, parakeratosis (p - 0.038), Munro’s microabscesses (p - 0.001), supra-papillary plate thinning (p - 0.004), and increased dermal vasculature (p - 0.001) were significantly associated with psoriasis when compared with psoriasiform dermatitis. Most psoriatic lesions had hypogranulosis whereas it was alternating hypo and hypergranulosis in psoriasiform dermatitis (p - 0.001). Acanthosis was regular in psoriasis lesions (p - 0.036). Bcl-2 expression was seen in dermal lymphocytes in all psoriasis and psoriasiform lesions with significantly stronger positive expression compared to basal and supra-basal layers. Psoriasis cases demonstrated more significant Bcl-2 positivity in dermal lymphocytes and basal layers than psoriasiform dermatitis cases.

Conclusion

Bcl-2 has stronger expression in dermal lymphocytes and basal layers than supra-basal layers, which comply with the proposed pathogenesis. Increased Bcl-2 expression in psoriasis cases compared with other psoriasiform dermatitis lesions implies the more chronic and recurrent nature of the disease.

## Introduction

Psoriasis is a chronic inflammatory cutaneous disorder that exhibits epidermal changes accompanied by markedly increased dermal vasculature. Its prevalence is about 2% of the world’s total population. Apart from cutaneous inflammation, psoriasis is characterized by significant epidermal hyperplasia and hyperproliferation along with increased dermal vascularity [1, 2].

The complex pathogenesis behind psoriasis involves an interplay of environmental, genetic, and immune-related factors, which is not completely understood yet. The recent supposed pathogenesis of psoriasis involves various cytokines like interleukin (IL)-1, IL-8, tumor necrosis factor (TNF-alpha), interferon (IFN-gamma), transforming growth factor (TGF-alpha), and others. These cytokines are produced by stimulated keratinocytes in a genetically predisposed individual, which leads to a series of pathogenetic steps. Upon endothelial activation, lymphocytes are recruited, which further amplifies inflammatory mechanisms by keratinocyte-lymphocyte interaction ultimately leading to the hyperproliferation of keratinocytes [3].

Keratinocytes are regulated by cell division, maturation, and death to maintain the structural homeostasis of normal skin. Cell deletion is governed by apoptosis, the physiological death of cells in a programmed manner. As epidermal thickening is evident in psoriasis skin, a possible abnormal apoptotic process-related homeostasis imbalance is indicated [4].

The Bcl-2 protein family regulates apoptosis. Among those, Bcl-xl along with Bcl-2 prevents apoptosis, while Bax, Bak, and Bid induce it. Studies noted that the keratinocytes in psoriasis lesions have an increased resistance to the apoptotic process, which could be a main mechanism in pathogenesis [5].

Although the Bcl-2 expression and its role in the pathogenesis of psoriasis was studied previously, many studies provided controversial results. Bcl-2 expression was observed in normal basal keratinocytes, whereas it was negative in the epidermis of psoriasis patients, according to one study [6]. As per another study, neither normal nor psoriasis epidermis expressed Bcl-2 [7]. Another study showed Bcl-2 expression in basal and spinous cell layers [4]. Further research studied the significant expression of Bcl-2 in lymphocytes in the dermis of psoriasis lesions compared to non-psoriatic lesions. These results suggested the pathogenetic basis for psoriasis’s chronicity, recurrence, and inflammatory character [8]. As we see, the comparison analysis of the expression of Bcl-2 in different compartments of skin and the possible underlying pathogenesis was not studied much.

Psoriasiform dermatitis involves a group of conditions which include lichen simplex chronicus, pityriasis rubra pilaris, inflammatory linear verrucous epidermal nevus (ILVEN), and unclassified forms [9]. Though the underlying pathogenesis may differ, differentiating psoriasis from psoriasiform dermatitis is always challenging because of the overlapping histopathological features. The level of expression of Bcl-2 and the underlying pathophysiology in these conditions were not studied much.

Hence, we aim to analyze the immunohistochemical (IHC) expression of Bcl-2 in different skin compartments in psoriasis as well as psoriasiform dermatitis lesions in this study.

## Materials and methods

We performed an analytical cross-sectional study on the paraffin blocks of psoriasis and psoriasiform dermatitis patients between 2022 and 2023 at our institute.

Inclusion and exclusion criteria

Clinically suspected and histologically diagnosed cases of psoriasis and psoriasiform dermatitis were included in the study. Psoriasis patients with previous treatment history, and currently either treated or in recurrence or relapse were excluded from the study. Patients with other co-existing dermatological conditions, or with any systemic illnesses known to affect the skin were also excluded from the study.

Staining technique

Skin biopsy specimens were received in 10% buffered formalin. Proper fixation, grossing, tissue processing, and section cutting were done per standard protocol. For histopathological examination (HPE), the sections were stained by hematoxylin and eosin (H&E). For old cases, patient details were collected from the case sheets. Tissue paraffin blocks and H&E-stained slides were obtained from the department archive. For Bcl-2 IHC staining, additional sections were cut from the paraffin block of tissue and were taken on a glass slide coated with adhesive aminopropyltriethoxysilane (APTES).

Primary antihuman antibody against Bcl-2 (rabbit monoclonal antibody from path-in-situ) was used. Nuclear along with cytoplasmic expression was considered positive for Bcl-2. The stained epidermal keratinocytes were categorized as supra-basal and basal cells. Lymphocytes in the dermis were also examined and graded.

Scoring method

The positively stained cells were scored at ×400 magnification. In dermal lymphocytes, semi-quantitative estimation of Bcl-2 expression was done by a scoring system devised by Yildiz et al. As per that, scores of 0 (no staining of lymphocytes), 1+ (staining less than 25%), 2+ (staining between 26 and 50%), 3+ (between 51 and 75%), and 4+ (over 75% staining) were assigned [8]. As for keratinocytes, the percentage of positive cells stained was classified as follows: 0 negative (if less than 5% stained); 1+ weakly positive (if 5-25% stained); 2+ moderately positive (if 25-50% stained); 3+ strongly positive (if more than 50% of cells stained) [10]. In this study, a cut-off of more than 25% of stained cells was considered positive and analyzed [11].

Statistical analysis

Statistical analysis was performed using Microsoft Excel and SPSS Statistics version 19.0. Continuous variables were summarized as mean and standard deviation (SD) or median (interquartile range), whereas categorical variables were presented as frequencies and percentages. The results were analyzed using the chi-square test. P-value < 0.05 is considered statistically significant.

## Results

Histopathological features of psoriasis and psoriasiform dermatitis

Thirty cases of psoriasis and 30 psoriasiform dermatitis cases were included. The differences in the HPE features between these two conditions were studied and given in Table 1. It was noted that parakeratosis was present in all cases of psoriasis vulgaris (p - 0.038). Munro’s microabscesses (p - 0.001), supra-papillary plate thinning (p - 0.004), and increased dermal vasculature (p - 0.001) were significantly associated with psoriasis when compared with psoriasiform dermatitis. The granular layer was found to be hypogranular in the majority of the psoriasis lesions, whereas it was alternating hypo and hypergranular in psoriasiform dermatitis cases (p - 0.001). Acanthosis was found to be regular in psoriasis lesions whereas there was irregularly formed acanthosis in psoriasiform dermatitis cases (p - 0.036).

**Table 1:** HPE features of psoriasis and psoriasiform dermatitis cases (n=60) The differences in the HPE features among psoriasis and psoriasiform dermatitis cases analyzed by the chi-square test are shown here. HPE: histopathological examination

HPE features		Psoriasis (n = 30)	Psoriasiform dermatitis (n = 30)	P-value
Hyperkeratosis	Yes	29 (96.6%)	30 (100%)	0.313
	No	1 (3.4%)	0	
Parakeratosis	Yes	30 (100%)	26 (86.6%)	0.038
	No	0	4 (13.4%)	
Granular layer	Normal	4 (13.4%)	3 (10%)	0.001
	Hyper	1 (3.3%)	11 (36.6%)	
	Hyper and hypo	6 (20%)	14 (46.6%)	
	Hypo	19 (63.3%)	2 (6.8%)	
Munro’s microabscesses	Yes	27 (90%)	11 (36.6%)	0.001
	No	3 (10%)	19 (63.4%)	
Acanthosis	Regular	23 (76.6%)	19 (63.4%)	0.036
	Irregular	4 (13.4%)	11 (36.6%)	
	No	3 (10%)	0	
Spongiosis	Yes	11 (36.6%)	12 (40%)	0.791
	No	19 (63.3%)	18 (60%)	
Supra-papillary plate	Normal	10 (33.4%)	21 (70%)	0.004
	Thinned	20 (66.6%)	9 (30%)	
Dermal vasculature	Yes	28 (93.4%)	9 (30%)	0.001
	No	2 (6.6%)	21 (70%)	

Bcl-2 and psoriasis

**Figure 1:**
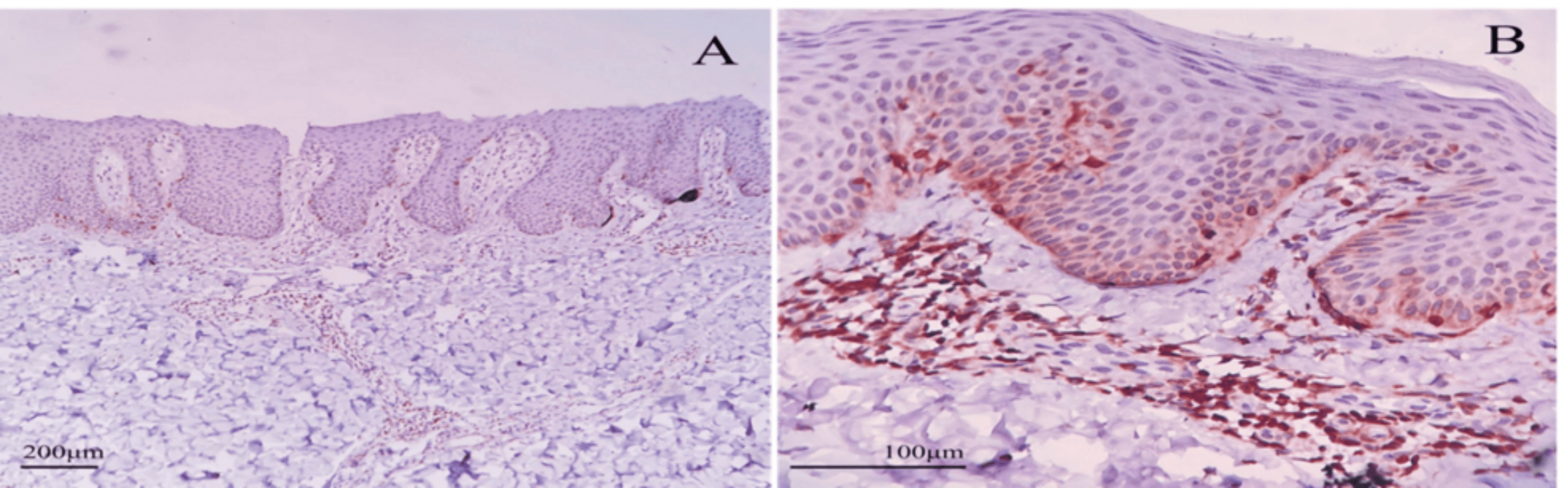
Immunohistochemical (IHC) examination: Bcl-2 expression in psoriasis IHC examination of psoriasis in (A) 100× magnification and (B) 400× magnification showing nuclear and cytoplasmic expression of Bcl-2 predominantly in dermal lymphocytes followed by basal keratinocyte layer and supra-basal layer.

IHC staining of Bcl-2 was observed and epidermal keratinocytes were categorized as supra-basal and basal layer cells and dermal lymphocyte staining was noted. Bcl-2 was evaluated, and quantification was done as mentioned earlier. It was observed that Bcl-2 was moderate to strongly positive in dermal lymphocytes, with moderate positivity in basal keratinocytes and no or weak staining in supra-basal keratinocytes in most cases as shown in Figure 1. From this, we interpreted that there was significant staining of Bcl-2 in the lymphocytes of the dermis compared to basal and supra-basal epidermal keratinocytes with 73%, 56%, and 17% of cells stained, respectively (p - 0.001).

Comparison of Bcl-2 positivity among cases of Psoriasis and Psoriasiform dermatitis

**Figure 2:**
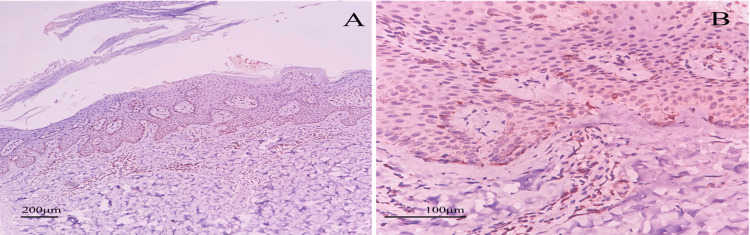
Immunohistochemical (IHC) examination of Bcl-2 in psoriasiform dermatitis IHC examination of psoriasiform dermatitis in (A) 100× magnification and (B) 400× magnification shows a nuclear and cytoplasmic expression of Bcl-2 more in dermal lymphocytes compared to basal keratinocyte layer and supra-basal layer.

**Figure 3:**
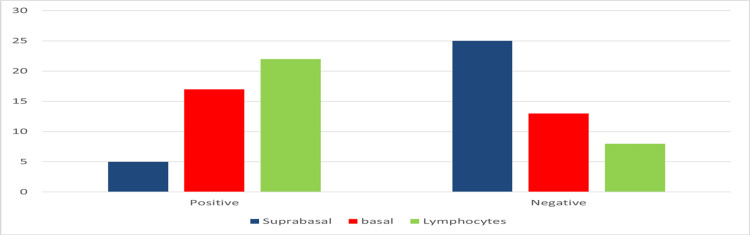
Comparision of Bcl-2 staining in psoriasis and psoriasiform dermatitis (n=60) Bcl-2 expression in psoriasis and psoriasiform dermatitis showing predominant staining in dermal lymphocytes followed by basal keratinocytes and supra-basal keratinocytes. The overall expression of Bcl-2 is found to be more in psoriasis than in psoriasiform dermatitis.

Bcl-2 positivity and its distribution among different compartments were compared among cases of psoriasis and psoriasiform dermatitis. Psoriasiform dermatitis cases were found to have a similar pattern of distribution of Bcl-2 as in psoriasis cases (Figure 2), but these cases had mainly weak to moderate positivity compared to psoriasis cases which had mainly moderate to strong positivity of Bcl-2 staining. Using >25% staining as the cut-off for positivity, it was clearly shown that basal keratinocytes and dermal lymphocytes have significant Bcl-2 positivity compared to similar areas in psoriasiform cases. The results are shown in Table 2 and Figure 3.

**Table 2:** Comparison of Bcl-2 staining in psoriasis and psoriasiform dermatitis (n=60) Staining >25% of cells is used as the cut-off for positivity. Analysis by chi-square test clearly showed that basal keratinocytes and dermal lymphocytes have significant Bcl-2 positivity compared to similar areas in psoriasiform cases. HPE: histopathological examination

HPE features		Psoriasis (n=30)	Psoriasiform dermatitis (n=30)	P-value
Supra-basal	Positive	5 (16.6%)	3 (10%)	0.448
	Negative	25 (83.4%)	27 (90%)	
Basal	Positive	17 (56.6%)	7 (23.4%)	0.018
	Negative	13 (43.4%)	23 (76.6%)	
Lymphocytes	Positive	22 (73.4%)	8 (26.6%)	0.001
	Negative	8 (26.6%)	22 (73.4%)	

## Discussion

Apoptosis was described for the first time by Kerr et al. as an inherently active programmed cell death that helps maintain skin homeostasis. Morphologically, apoptosis is characterized by nuclear condensation and fragmentation of cells followed by phagocytosis by dendritic cells and macrophages [12]. Apoptosis in the keratinocytes helps in balancing its proliferation to maintain the epidermal thickness, in the formation of stratum corneum, and may also help to eliminate pre-malignant cells [13].

The epidermal change in psoriasis includes hyperproliferation and incomplete differentiation of keratinocytes [14]. There are various pathogenetic mechanisms proposed for the development of psoriasis. A significant pathogenetic mechanism was found to be an enhanced ability of the psoriasis keratinocytes to resist apoptosis [15]. As discussed earlier, the Bcl-2 protein family regulates apoptosis. Among those, proteins that block apoptosis like Bcl-2 are studied most. There are many studies done in the past, which tried to understand the Bcl-2 expressivity in different compartments of psoriasis epidermis and dermis and the likely underlying pathogenetic mechanisms.

Earlier, Bianchi et al. demonstrated a drastic reduction of Bcl-2 expressivity in the basal compartment of psoriasis lesions compared to uninvolved normal skin. The reason behind this was extrapolated as the involvement of undifferentiated cells in these conditions [6]. Later an observation study by Wrone-Smith et al. reported that Bcl-2 was not expressed in either normal or psoriasis epidermis [7]. Takahashi et al. stated that these studies used antihuman Bcl-2 antibodies (monoclonal mouse 124; DAKO) and then studied with anti-rabbit Bcl-2 antibody (rabbit polyclonal sc-783; Santa Cruz) and demonstrated the graded expression of Bcl-2 from basal-to-spinous cell layers of the epidermal keratinocytes. By mRNA-based analysis, they showed that the uninvolved epidermis had higher Bcl-2 expression than the involved epidermis [4]. In addition, many studies that compared psoriasis skin with normal skin have reported the decreased expression of Bcl-2 in psoriasis keratinocytes [16-19]. In our study also, we found graded positivity of Bcl-2, with stronger expression in basal layers compared to supra-basal layers. The results were similar in the cases of psoriasiform dermatitis. These results are compliant with the previous results as the keratinocytes are formed in the basal layer and ascend upward. This increased anti-apoptotic activity in the basal layers compared to supra-basal layers forms the basis of one of the pathogenetic mechanisms in psoriasis. The dynamic nature of the disease was explained in literature by decreased expression of Bcl-2 in psoriasis compared to normal skin, which was not studied in this study.

Later many studies have demonstrated the significantly increased expression of Bcl-2 in the lymphocytes of psoriasis skin dermal layers [8, 20]. Lymphocytes of the dermis with increased expressivity of Bcl-2 were attributed to increased lymphocyte survival and continuous and regular cytokine secretion. The chronicity and recurrence of this disease were then mainly explained by this mechanism. Emerging evidence supports this fact and the role of memory T cells. Once antigen-presenting cells activate naive T cells, a few of them differentiate into precursor memory T cells. These cells ultimately form many subsets of memory T cells, one of which are tissue-resident memory T (TRM) cells. TRM cells, under physiological conditions, reside in the skin and can respond rapidly to pathogenic challenges. There is evidence to support the vital role of TRM cells in the recurrence nature of chronic inflammatory skin disorders like psoriasis [21]. In our study, we have used a primary antihuman antibody against Bcl-2 (rabbit monoclonal antibody from path-in-situ), and we have analyzed the expressivity of Bcl-2 in different compartments of psoriasis and psoriasiform dermatitis skin lesions. We found that Bcl-2 expression was present in almost all cases of psoriasis and psoriasiform lesions. Moreover, we found that dermal lymphocytes showed moderate to stronger staining of Bcl-2 compared to basal and supra-basal layers, complying with the earlier results and attributed pathogenesis.

We also found the association of Bcl-2 positivity among cases of psoriasis and psoriasiform lesions. Psoriasiform dermatitis mimics psoriasis either clinically or histologically and must be distinguished from psoriasis since the treatment differs for both. This category includes unclassified psoriasiform dermatitis, Lichen simplex chronicus, Pityriasis rubra pilaris, ILVEN, and others [9, 22]. Similar findings were also reported in cases of lichen planus [23, 24]. None of the previous studies have directly studied the association of Bcl-2 in psoriasiform dermatitis compared with psoriasis. In our study, we recruited 30 cases of psoriasiform dermatitis in which two cases were Lichen simplex chronicus and rest were unclassified forms. We noted that Bcl-2 was more strongly expressed in basal and dermal lymphocytes of psoriasis cases than in psoriasiform dermatitis cases, which could probably explain the more aggressive, chronic, and recurrent nature of psoriasis compared to other psoriasiform dermatitis lesions. This could probably form the basis of treatment by anti-Bcl-2 antibodies in more chronic and recurrent cases of psoriasis.

By using the criteria as >25% of total cells stained as positive [11], we found that basal and dermal lymphocytes showed significant Bcl-2 positivity than supra-basal layers in both conditions. It was also noted that psoriasis lesions were significantly positive compared to psoriasiform dermatitis cases. The difference in treatment protocols for psoriasiform dermatitis [25-27] and psoriasis [28-30] is markedly significant. As there is a histopathological overlap of features in psoriasis and psoriasiform dermatitis cases, we suggest that Bcl-2 can be used as a marker in differentiating these conditions for accurate diagnosis and targeted therapeutic approaches.

Limitations

Our study has certain limitations. First, we haven’t analyzed Bcl-2 expression in different forms of psoriasis, as it may show differences in different morphological patterns. Correlation with clinical severity of psoriasis and the association with Psoriasis Area and Severity Index (PASI) scores were not done. Second, since psoriasis is a dynamic disease, the level of expression of Bcl-2 may vary in different stages of the disease. In our study, we haven’t analyzed the stage of the disease. Third, since our study majorly has unclassified forms of psoriasiform dermatitis, we couldn’t analyze the level of Bcl-2 expression in individual subtypes due to skewed distribution. So, further studies with a greater number of patients along with clinicopathological correlation will provide much insight into the expression of Bcl-2 in these cases and the underlying pathogenesis.

## Conclusions

Studies with anti-apoptotic factor Bcl-2 expression in psoriasis provided controversial results. We found that Bcl-2 expression is stronger in dermal lymphocytes than keratinocytes, and also more in basal regions compared to supra-basal areas. We also report that Bcl-2 shows more significant positivity in basal keratinocytes and dermal lymphocytes in psoriasis lesions than in psoriasiform dermatitis cases. More strong expression in psoriasis cases compared with other psoriasiform dermatitis lesions implies the more chronic and recurrent nature of the disease. So, we suggest that Bcl-2 can be used as a marker in differentiating these conditions for accurate diagnosis and targeted therapeutic approaches.

## References

[1] Mobini N (2005). Noninfectious erythematous, papular, and squamous disease. Lever's Histopathology of the Skin.

[2] Ramezani M, Shamshiri A, Zavattaro E, Khazaei S, Rezaei M, Mahmoodi R, Sadeghi M (2019). Immunohistochemical expression of P53, Ki-67, and CD34 in psoriasis and psoriasiform dermatitis. Biomedicine (Taipei).

[3] Bonifati C, Ameglio F (1999). Cytokines in psoriasis. Int J Dermatol.

[4] Takahashi H, Manabe A, Ishida-Yamamoto A, Hashimoto Y, Iizuka H (2002). Aberrant expression of apoptosis-related molecules in psoriatic epidermis. J Dermatol Sci.

[5] Kaštelan M, Prpić-Massari L, Brajac I (2009). Apoptosis in psoriasis. Acta Dermatovenerol Croat.

[6] Bianchi L, Farrace MG, Nini G, Piacentini M (1994). Abnormal Bcl-2 and "tissue" transglutaminase expression in psoriatic skin. J Invest Dermatol.

[7] Wrone-Smith T, Johnson T, Nelson B (1995). Discordant expression of Bcl-x and Bcl-2 by keratinocytes in vitro and psoriatic keratinocytes in vivo. Am J Pathol.

[8] Yildiz L, Bariş S, Senturk N, Kandemir B (2003). Overexpression of bcl‐2 in lymphocytes of psoriatic skin. J Eur Acad Dermatol Venereol.

[9] Patterson JW (2014). Weedon's Skin Pathology E-book.

[10] Nakagawa K, Yamamura K, Maeda S, Ichihashi M (1994). Bcl‐2 expression in epidermal keratinocytic diseases. Cancer.

[11] Ramezani M, Hashemi BS, Khazaei S, Rezaei M, Ebrahimi A, Sadeghi M (2017). Diagnostic value of immunohistochemistry staining of Bcl-2, CD34, CD20 and CD3 for distinction between discoid lupus erythematosus and lichen planus in the skin. Indian J Pathol Microbiol.

[12] Kerr JF, Wyllie AH, Currie AR (1972). Apoptosis: A basic biological phenomenon with wide-ranging implications in tissue kinetics. Br J Cancer.

[13] Raj D, Brash DE, Grossman D (2006). Keratinocyte apoptosis in epidermal development and disease. J Invest Dermatol.

[14] McKay IA, Leigh IM (1995). Altered keratinocyte growth and differentiation in psoriasis. Clin Dermatol.

[15] Wrone-Smith T, Mitra RS, Thompson CB, Jasty R, Castle VP, Nickoloff BJ (1997). Keratinocytes derived from psoriatic plaques are resistant to apoptosis compared with normal skin. Am J Pathol.

[16] Gündüz K, Demireli P, Vatansever S, Inanir I (2006). Examination of bcl-2 and p53 expressions and apoptotic index by TUNEL method in psoriasis. J Cutan Pathol.

[17] Batinac T, Zamolo G, Hadžisejdić I (2007). Expression of Bcl-2 family proteins in psoriasis. Croat Med J.

[18] Zou A, Chen Y, Liu T, Yang T, Zhou B (2023). Identification and verification of three autophagy-related genes as potential biomarkers for the diagnosis of psoriasis. Sci Rep.

[19] Koçak M, Bozdogan O, Erkek E, Atasoy P, Birol A (2003). Examination of Bcl-2, Bcl-X and bax protein expression in psoriasis. Int J Dermatol.

[20] Bal N, Tuncer I, Baba M, Bolat F (2008). Bcl-2 expression in dermal lymphocytes in lichen planus and psoriasis vulgaris. J Eur Acad Dermatol Venereol.

[21] Chen L, Shen Z (2020). Tissue-resident memory T cells and their biological characteristics in the recurrence of inflammatory skin disorders. Cell Mol Immunol.

[22] Elder DE, Elenitsas R, Murphy GF (202231). Lever's Dermatopathology: Histopathology of the skin.

[23] Arreaza AJ, Rivera H, Correnti M (2014). Expression of COX-2 and bcl-2 in oral lichen planus lesions and lichenoid reactions. Ecancermedicalscience.

[24] Leyva-Huerta ER, Ledesma-Montes C, Rojo-Botello RE, Vega-Memije E (2012). P53 and bcl-2 immunoexpression in patients with oral lichen planus and oral squamous cell carcinoma. Med Oral Patol Oral Cir Bucal.

[25] Juarez MC, Kwatra SG (2021). A systematic review of evidence based treatments for lichen simplex chronicus. J Dermatolog Treat.

[26] Klein A, Landthaler M, Karrer S (2010). Pityriasis rubra pilaris: A review of diagnosis and treatment. Am J Clin Dermatol.

[27] Renner R, Colsman A, Sticherling M (2008). ILVEN: is it psoriasis? Debate based on successful treatment with etanercept. Acta Derm Venereol.

[28] Armstrong AW, Read C (2020). Pathophysiology, clinical presentation, and treatment of psoriasis: A review. JAMA.

[29] Kaushik SB, Lebwohl MG (2019). Psoriasis: Which therapy for which patient: Focus on special populations and chronic infections. J Am Acad Dermatol.

[30] Rendon A, Schäkel K (2019). Psoriasis pathogenesis and treatment. Int J Mol Sci.

